# EhVps23, an ESCRT-I Member, Is a Key Factor in Secretion, Motility, Phagocytosis and Tissue Invasion by *Entamoeba histolytica*

**DOI:** 10.3389/fcimb.2022.835654

**Published:** 2022-03-14

**Authors:** Ausencio Galindo, Rosario Javier-Reyna, Guillermina García-Rivera, Cecilia Bañuelos, Bibiana Chávez-Munguía, Lizbeth Salazar-Villatoro, Esther Orozco

**Affiliations:** ^1^ Departamento de Infectómica y Patogénesis Molecular, Centro de Investigación y de Estudios Avanzados del Instituto Politécnico Nacional (IPN), Ciudad de México, Mexico; ^2^ Programa Transdisciplinario en Desarrollo Científico y Tecnológico para la Sociedad, Centro de Investigación y de Estudios Avanzados del Instituto Politécnico Nacional (IPN), Ciudad de México, Mexico

**Keywords:** EhVps23, phagocytosis, migration, secretion, invasion, *Entamoeba histolytica*

## Abstract

The EhVps23 protein, an orthologue of the yeast Vps23 and the mammalian TSG101 proteins, is the single member of the ESCRT-I complex of *Entamoeba histolytica* identified and characterized until now. EhVps23 actively participates in vesicular trafficking and phagocytosis, which influence several cellular events. In this paper, we investigated the role of EhVps23 in virulence-related functions, including the invasive capacity of trophozoites, using transfected trophozoites. Trophozoites overexpressing the EhVps23 protein (Neo-EhVps23) presented helical arrangements in the cytoplasm, similar to the ones formed by EhVps32 for scission of vesicles. By confocal and transmission electron microscopy, EhVps23 was detected in multivesicular bodies, vesicles, and the extracellular space. It was secreted in vesicles together with other proteins, including the EhADH adhesin. Probably, these vesicles carry molecules that participate in the prey capture or in cell-cell communication. Mass spectrometry of precipitates obtained using α-EhVps23 antibodies, evidenced the presence of proteins involved in motility, phagocytosis, vesicular trafficking and secretion. The study of cellular functions, revealed that Neo-EhVps23 trophozoites exhibit characteristics similar to those described for mammalian transformed cells: they grew 50% faster than the control; presented a significant higher rate of phagocytosis, and migrated five-fold faster than the control, in concordance with the low rate of migration exhibited by *Ehvps23*-knocked down trophozoites. In addition, Neo-EhVps23 trophozoites produced dramatic liver abscesses in experimental animals. In conclusion, our results showed that EhVps23 overexpression gave to the trophozoites characteristics that resemble cancer cells, such as increased cell proliferation, migration, and invasion. The mutant that overexpresses EhVps23 can be a good study model to explore different events related to the transformation of malignant cells.

## Introduction

Several studies have identified the genes and proteins required for endosomal sorting in mammalian cells. However, even when these are fundamental for nutrition, and capturing and internalization of the prey, they remain poorly known in *Entamoeba histolytica*. This protozoan causes human amoebiasis, affecting 50 million (Bercu et al., 2007) and killing 100,000 individuals around the world each year (World Health Organization, 1997). In our efforts to understand the molecular basis of the aggressive mechanism of the trophozoites, we have studied proteins of the endosomal sorting complexes required for transport (ESCRT) (Avalos-Padilla et al., 2015; Avalos-Padilla et al., 2018; Galindo et al., 2021), and others associated that cooperate in molecular trafficking and virulence events of this parasite (Lopez-Reyes et al., 2010; Bañuelos et al., 2012).

We know that the *E. histolytica* ESCRT machinery possesses at least 19 genes (Lopez-Reyes et al., 2010), whose products act in several steps of endosomal sorting, vesicular trafficking and phagocytosis (Lopez-Reyes et al., 2010; Bañuelos et al., 2012; Avalos-Padilla et al., 2015; Avalos-Padilla et al., 2018; Galindo et al., 2021). So far, we have found that the ESCRT-III complex (Avalos-Padilla et al., 2015; Avalos-Padilla et al., 2018) proteins work together and with the EhADH protein, an ALIX family member (Bañuelos et al., 2012), and the EhVps4 ATPase (Lopez-Reyes et al., 2010), the enzyme required to maintain the cycle of binding and release of ESCRT-III proteins from the endosomal membranes. Meanwhile, EhVps23 (ESCRT-I) interacts with EhADH, the lysobisphosphatidic acid (LBPA) and EhUbiquitin (Galindo et al., 2021).

Yeast Vps23 and mammalian TSG101 proteins form part of the tetrameric conserved ESCRT-I complex in both systems (Kostelansky et al., 2007; Flower et al., 2020). In contrast, EhVps23, the *E. histolytica* orthologue is the unique member of this complex characterized until now in the parasite (Galindo et al., 2021). It is predominantly a cytosolic protein, but when the trophozoites undergo phagocytosis, EhVps23 is ubiquitinated and appears close to the erythrocytes and in the endosomes, phagosomes and multivesicular bodies (MVBs). Co-immunoprecipitation and immunofluorescence microscopy experiments have shown that it interacts directly or indirectly with EhADH (Galindo et al., 2021). In addition, structural and docking analysis predicted that the contact of EhVps23 with EhADH, EhUbiquitin, and EhVps32 proteins is through the amino terminus, whereas it contacts the LBPA by the C-terminal region, possibly generating a multiprotein complex.

The knock down of the *Ehvps23* gene reduces cell proliferation and phagocytosis (Galindo et al., 2021), evidencing the role of this protein in the parasite virulence. Multiprotein complexes formed by Vps23 and TSG101 are involved in many cellular functions, such as endosomal sorting, receptor internalization, ligand uptake, protein transferring to MVBs, cell division, and others (Szymanska et al., 2018; Vietri et al., 2020). Furthermore, some authors have suggested that TSG101 might recognize cargo destined to the MVBs, or as a structural component for molecular sorting events (Bishop and Woodman, 2001; White et al., 2017), Interestingly, TSG101 possesses DNA binding motifs, which would allow this protein to participate as a transcriptional suppressor of certain genes (Lin et al., 2013). In this context, TSG101 could act not only in the endosomal sorting and protein transport (Szymanska et al., 2018), but they could participate in regulation of genes involved in functions such as motility and tissue invasion (Chua et al., 2019), and it is plausible that the EhVps23 orthologues displays similar functions in the trophozoites.

Here, we present data that evidence that trophozoites overexpressing EhVps23 grow and move faster than the control, they presented a discrete, but significant increase in phagocytosis, and a dramatic capacity to produce hepatic abscesses in experimental animals. In contrast, when the *Ehvps23* gene is silenced, the migration capacity is decreased, pointing out that EhVps23 has indeed a role in motility.

## Materials and Methods

### *E. histolytica* Cultures

*E. histolytica* trophozoites, strain HM1:IMSS, were axenically grown at 37°C in TYI‐S‐33 medium and harvested at logarithmic growth phase (Diamond et al., 1978). To harvest the trophozoites, the culture flasks were incubated 10 min at 4°C. Medium for transfected trophozoites was supplemented with G418 (Gibco). All experiments presented here were performed at least three times by duplicate.

### Antibodies

For EhADH immunodetection, we obtained hamster polyclonal antibodies (α-EhADH) against a specific EhADH peptide (N_566_-QCVINLLKEFDNTKNI-C_582_) localized within the adherence domain. Male hamsters were immunized three times (each 2 weeks) with 300 μg of this peptide diluted in TiterMax^®^ Gold Adjuvant liquid (Sigma), then, animals were bled and antibodies obtained. Other primary antibodies used were: rat α-EhVps23 (Galindo et al., 2021), mouse monoclonal α-HA (GeneTex), mouse α-EhVps32 (Avalos-Padilla et al., 2015), mouse monoclonal α-Ubiquitin (Santacruz), rabbit α-EhCP112 (García-Rivera et al., 1999) and mouse monoclonal α-human actin (kindly given by Dr. Manuel Hernández, Cell Biology Department, CINVESTAV) antibodies. Secondary antibodies were: HRP-labelled α-rabbit, α-mouse, α-hamster and α-rat IgGs (Zymed) for western blot. FITC-labelled α-rabbit and α-mouse IgGs, Alexa 647 α-hamster and TRITC-labelled or Alexa 405 α-rat IgGs (Life Technologies) for immunofluorescence. For immunoelectron microscopy experiments, we used α-rat IgG, conjugated with 10 nm gold particles (TED Pella Inc).

### RT-PCR Assays

Total RNA was isolated using TRIzol reagent (Invitrogen). Complementary DNA (cDNA) was synthesized using oligo dT primers and the Superscript II reverse transcriptase (Invitrogen). PCR amplifications were carried out using Q5 ^®^ High-Fidelity DNA Polymerase (Biolabs) according to manufacturer’s recommendations with 20 ng of cDNA as templates, and specific primers. Products were separated by electrophoresis in 1% agarose gels and stained with ethidium bromide. As control for PCR amplifications, instead of cDNA, nuclease-free water was used.

### Plasmid Constructs

For overexpression experiments the *Ehvps23_1123-1485 bp_* or *Ehvps23 *full length sequences were PCR-amplified using cDNA as template and specific primers. For *Ehvps23_1123-1485 bp_*: sense 5′-GGGGTACCATGGAAGAGTCTGAAGAAATACTTCATG-3′, and antisense 5′-CCGGATCCTTATTCAGTTATGCAATACTTTGCATGAA-3′; for *Ehvps23 *full length sense 5′-AAGGTACCATGTACCCATATGATGTTCCAGATTATGCTCAACCTATAAACAATGAAAA-3′, and antisense 5′-CCGGATCCTTATTCAGTTATGCAATACTTTGCATGAAATTGAATTGAAGTTAT-3 oligonucleotides. Underlined sequences correspond to the enzyme restriction sites. Fragments were cloned into the *pNeo* plasmid, generating *pNeoEhvps23_1123-1485 pb_* and *pNeoEhvps23*, plasmids, respectively, both labeled with an hemagglutinin (HA) tag. The constructs were verified by restriction analyses and automatic DNA sequencing.

### Transfection Assays

Trophozoites were transfected as previously described (Hamann et al., 1995). Briefly, parasites were grown in 35-mm Petri dishes overnight and transfected with 20 μg of the *pNeoEhvps23*, *pNeoEhvps23_1123-1485 pb_ or pNeo* plasmid, using the SuperFact reagent (Qiagen). Transfected trophozoites were grown for 48 h at 37°C in TY1-S medium after plus 40 μg/ml G418 and maintained as stable cell cultures (Neo-EhVps23 and Neo populations). EhVps23 overexpression in NeoEhVps23 trophozoites was confirmed by western blot and immunofluorescence assays.

### Western Blot Assays

Total extracts from trophozoites (prepared in the presence of 100 mM PHMB, 2.7 mM E64 and a cocktail of protease inhibitors) were separated by SDS-PAGE gels, transferred onto nitrocellulose membranes, and probed with rat α-EhVps23 (1:500), rabitt α-HA (1:3,000), mouse α-EhVps32 (1:500), mouse α-Ub (1:100), hamster α-EhADH (1:500), rabbit α-EhCP112 (1:3,000) or mouse α-actin (1:3,500) antibodies. Membranes were incubated with the species-specific horseradish peroxidase (HRP)-labelled secondary antibodies (1:10,000), and developed with the ECL Prime detection reagent (GE-Healthcare). Pre-immune serum was used as a control.

### Immunofluorescence Assays

Trophozoites grown on coverslips, were fixed with 4% paraformaldehyde at 37°C for 1 h, permeabilized with 0.2% Triton X‐100 and blocked with 10% fetal bovine serum in PBS. Then, preparations were incubated at 4°C overnight (ON) with EhVps23 (1:50), α-HA (1:300), α-EhADH (1:50) or α-EhCP112 (1:200) antibodies, followed by incubation for 30 min at 37°C with TRITC-labelled or Alexa 405 α-rat IgG for α-EhVps23, FITC-labelled α-rabbit IgG for α-EhCP112 and Alexa 647-labelled α-hamster IgG for α-EhADH. All preparations were preserved using the Vectashield antifade reagent (Vector). 0.5 µm laser sections were examined through a Carl Zeiss LMS 700 confocal microscope and processed with ZEN 2009 Light Edition Software (Zeiss).

### Growth Rate Assays

Trophozoites (0.2 × 10^5^ for Neo-EhVps23 or 2 × 10^5^ Neo-EhVps23c) cultured in TYI-S medium supplemented with 0 to 40 μg/ml of G418, were incubated at 37°C for several days. The number of trophozoites was counted and registered every 24 h and viability was evaluated by trypan blue exclusion using a 0.2% dye solution, and examined under the light microscope (Bañuelos et al., 2012).

### Scanning Electron Microscopy (SEM)

Samples were prepared for SEM as described (Avalos-Padilla et al., 2015). Briefly, glutaraldehyde-fixed samples were dehydrated with increasing concentrations of ethanol and CO_2_ critically point dried in a Samdri 780 apparatus (Tousimis Corp). Then, they were gold-coated in an ion sputtering device (Jeol-JFC-1100) and examined through a Jeol JSM-7100F field emission scanning electron microscope (JEOL Ltd).

### Transmission Electron Microscopy (TEM)

Samples were prepared for TEM as described (Bolaños et al., 2016). Briefly, trophozoites were fixed with 4% paraformaldehyde and 0.5% glutaraldehyde in PBS for 1 h at room temperature. Samples were embedded in LR White resin (London Resin Co) and polymerized under UV at 56°C ON. Thin sections (60 nm) were mounted on Formvar‐covered nickel grids followed by ON incubation with α‐EhVps23 (1:30), then, incubated ON with the secondary antibodies (1:50) conjugated to 10‐nm gold particles. Thin sections were contrasted with uranyl acetate and lead citrate and observed with a Joel JEM‐1011 transmission electron microscope (JEOL Ltd).

### Secretion Assays

Trophozoites (3 × 10^6^) were washed three times in PBS and incubated with 200 μl of PBS supplemented with 1 mg/ml of E64 (Sigma) and proteases inhibitor cocktail (Roche) for 2 h at 37°C. PBS was collected and centrifuged at 13,000 x *g* for 10 min to obtain the secreted molecules in the supernatant fraction. The trophozoites in the pellet were lysed in the presence of proteases inhibitors as reported (Bolaños et al., 2016). Samples were submitted to 10% SDS-PAGE and western blot assays using α-EhVps23, α-HA, α-EhVps32, α-EhADH, α-Ubiquitin, α-EhCP112 or α-actin antibodies as described above. In some experiments, the secretion products were fixed with glutaraldehyde (2%) to be analyzed by negative staining and TEM as previously described.

### Negative Staining

Negative staining was performed as previously described (Avalos-Padilla et al., 2015). The secretion products (5 μl) were pipetted onto the surface of the formvar-coated copper grids (400 mesh). Samples were blotted off with filter paper and stained with 2.5% uranyl acetate for 20 seconds. Grids were then left to air dry and carbon coated. Preparations were examined through a JEM-1011 transmission electron microscope.

### Purification of Extracellular Vesicles by Differential Centrifugation

The secretion products were processed to purify extracellular vesicles (Théry et al., 2006). Briefly, the secretion products were centrifuged at 10,000 x g for 30 minutes to remove cell debris. The final supernatant was then ultracentrifuged at 100,000 × g for 70 minutes to pellet small vesicles. The pellet was washed in one volume of PBS, to remove contaminating proteins, and centrifuged at the same high speed. The purified samples were analyzed by TEM as previously described.

### Immunoprecipitation Assays

Immunoprecipitation was performed using the α-EhVps23 as described (Galindo et al., 2021). Following the methodology used by Castellano Castro et al. (2016), trophozoites were lysed in the presence of 10 mM Tris‐HCl, 50 mM NaCl, and proteases inhibitors, by cycles of freeze‐thawing in liquid nitrogen and vortexing, then the lysates were pre-cleared with 200 µl of Protein-G (previously blocked with 2% BSA) and incubated 2 h at 4°C under gentle stirring. Subsequently immunoprecipitation assays were performed using 200 μl of protein G‐agarose (Invitrogen) and α-EhVps23 antibody. Bound proteins were identified in the Proteomics Unit of LaNSE (Laboratorio Nacional de Servicios Experimentales) at CINVESTAV.

### Mass Spectrometry Analysis LC-ESI-HDMSE

Each lane was sliced and enzymatically digested according to the modified protocol reported (Shevchenko et al., 2007). Afterward, tryptic peptides were loaded into Symmetry C18 Trap V/M precolumn (Waters); 180 μm × 20 mm, 100-A° pore size, 5-μm particle size, and desalted using as a mobile phase A (0.1% formic acid in H_2_O) and mobile phase B (0.1% formic acid in acetonitrile) under the following isocratic gradient: 99.9% mobile phase A and 0.1% of mobile phase B at a flow of 5 μl min−1 during 3 min. Then, peptides were loaded and separated on a HSS T3 C18 Column (Waters); 75 μm × 150 mm, 100-A° pore size, 1.8-μm particle size, using an UPLC ACQUITY M-Class (Waters) with the same mobile phases under the following gradient: 0 min 7% B, 30.37 min 40% B, 32.03–35.34 min 85% B, 37–47 min 7% B at a flow of 400 nl min^−1^ and 45°C. The spectra data were acquired in a mass spectrometer with electrospray ionization and ion mobility separation Synapt G2-Si (Waters) using data-independent acquisition approach through HDMSE mode (Waters). Generated *.raw files containing MS and MS/MS spectra were deconvoluted and compared using ProteinLynx Global SERVER (PLGS) v3.0.3 software (Li et al., 2009) against a reversed *Entamoeba histolytica* database (downloaded from Uniprot). All identifications had a reliability ≥ 95%.

### Migration Assays

Serum-starved (3 h) trophozoites (7.5 x 10^4^) were placed in the upper chamber of transwell insets (5 μm pore size, 24 well, Costar) and 500 μl of bovine serum were added to the lower chamber. Trophozoites were incubated for 3 h at 37°C and trophozoites migration was determined by counting the number of cells in the lower chamber of the transwell (Bolaños et al., 2016).

### *Ehvps23* Gene-Silencing Based on dsRNA

The knock-down of the *Ehvps23* gene was performed using double-stranded RNA (dsRNA) produced in bacteria transformed with the first 341 bp of the gene and introduced to the parasite by soaking experiments (Solis et al., 2009; Galindo et al., 2021). Briefly, the competent RNase III-deficient *E. coli* strain HT115 was transformed with the *pL4440Ehvps23_1-341 pb_*. Transformed bacteria were grown at 37°C in 2YT LB broth supplemented with ampicillin (100 mg/ml) and tetracycline (10 mg/ml). The expression of *Ehvps23*-dsRNA was induced with 2 mM (IPTG), ON at 37°C. Then, the bacterial pellet was mixed with 1 M ammonium acetate and 10 mM EDTA, incubated with phenol:chloroform:isoamyl alcohol (25:24:1) and centrifuged. The supernatant was mixed with isopropanol, centrifuged, and the nucleic acid pellet was washed with 70% ethanol. DNase I (Invitrogen) and RNase A (Ambion) were added to eliminate dsDNA and ssRNA molecules, respectively; *Ehvps23*-dsRNA was washed again with isopropanol and ethanol and analyzed by agarose gel electrophoresis; its concentration was determined by spectrophotometry. Lastly, purified *Ehvps23*-dsRNA molecules were added to the trophozoites (3.0 × 10^4^) in TYI-S-33 complete medium to a final concentration of 5 μg/ml in culture tubes of 5 ml, and cultures were incubated at 37°C for 72 h. Cells growing in standard conditions (without dsRNA) were used as controls.

### Phagocytosis Assays

For phagocytosis assays the trophozoites were incubated for 0, 15, 30 and 45 min with RBCs (1:25) at 37°C. At different times, trophozoites were prepared for immunofluorescence (García-Rivera et al., 1982) or stained by Novikoff technique (Novikoff et al., 1972); erythrocytes were counted in 100 trophozoites through the light microscope (Axiolab, Zeiss). In some experiments, trophozoites were lysed using absolute formic acid and the hemoglobin of ingested erythrocytes was quantified by spectrophotometry at 400 nm (Talamás-Lara et al., 2014).

### *In Vivo* Virulence Experiments

Four weeks-old male hamsters (*Mesocricetus auratus*) weighing 40 ± 5 g were fasted for 24 h prior to surgery. Subsequently, they were anesthetized with 3% isoflurane and then, with 1.5% of the same anesthetic during the surgical procedure. Abdominal surfaces of the hamsters were shaved, and a longitudinal incision of the abdominal wall was made, to expose the port vein and livers. 1.2 × 10^6^ trophozoites in 200 μl of TYI-S-33 without bovine serum were intraportally inoculated into the animals. Six days after the challenge, hamsters were sacrificed with an overdose of anesthetic; the whole liver was weighed, and then, the liver lesion was dissected and weighed to calculate the percentage of the damaged tissue in relation to the total liver weight (Pais-Morales et al., 2016).

### Statistical Analyses

Values for all experiments were expressed as the mean and standard error of at least three independent assays, carried out by duplicate. Statistical analyses were performed using the GraphPad Prism v5.01 software by a paired Student’s t test. *p < 0.05; **p < 0.01, and ***p < 0.001.

### Ethics Statement

CINVESTAV fulfills the standard of the Mexican Official Norm (NOM-062-ZOO-1999) “Technical Specifications for the Care and Use of Laboratory Animals”, based on the Guide for the Care and Use of Laboratory Animals (“The Guide” 2011, NRC, USA with the Federal Register Number BOO.02.03.02.01.908), awarded by the National Service for Agrifood Health, Safety and Quality (SENASICA). This organization verifies the state of compliance of such NOM in Mexico and belongs to the Ministry of Agriculture and Rural Development. The Institutional Committee for Animal Care and Use (IACUC/Ethics committee) from CINVESTAV, the regulatory office for research protocols approval involving the use of laboratory animals, reviewed, and approved all animal experiments (Protocol Number 0505-12, CICUAL 001).

## Results

### EhVps23 Influences the Rate of Growth of *E. histolytica* Trophozoites

**Figure 1:**
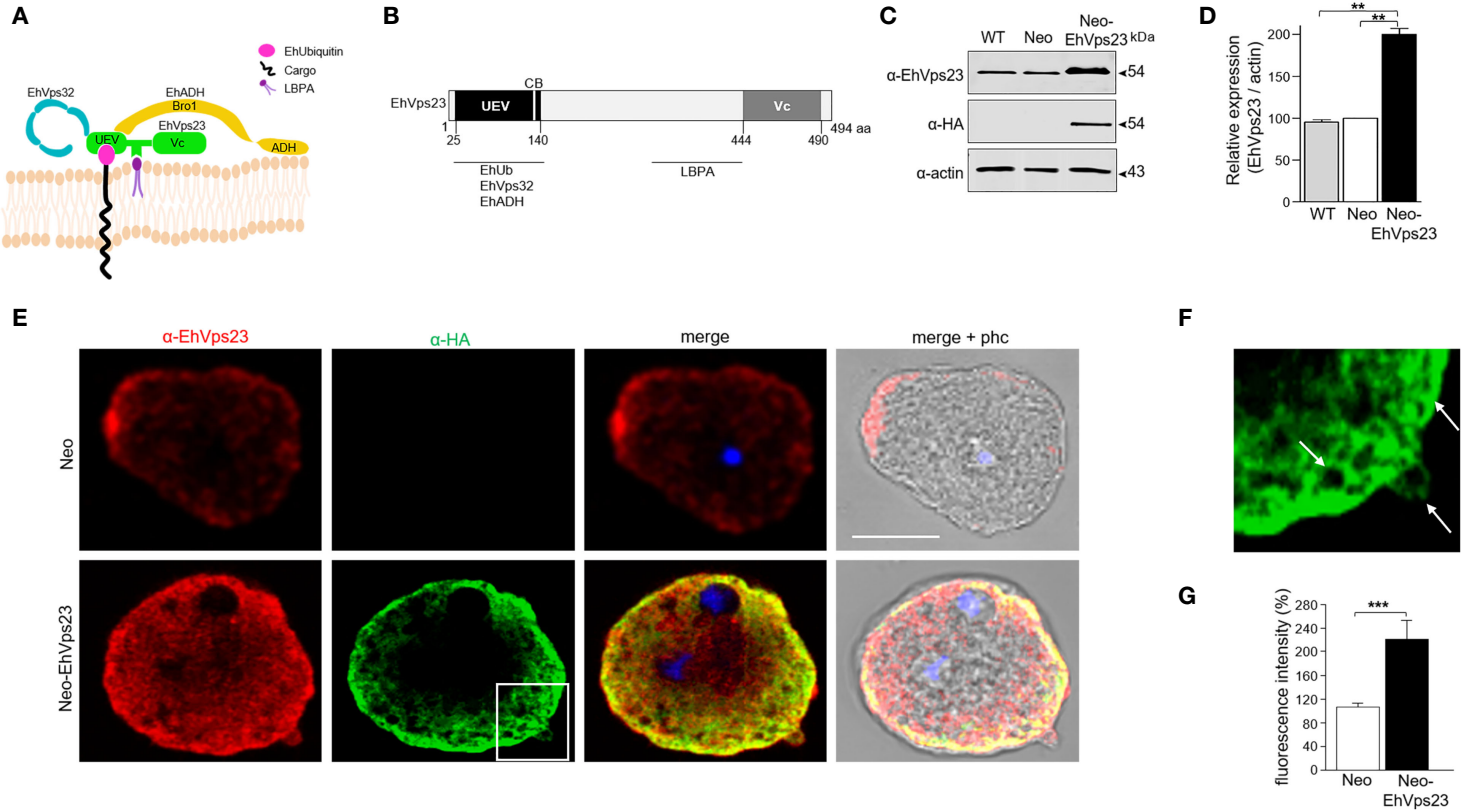
Generation of trophozoites overexpressing the EhVps23 protein. **(A)** Scheme of EhVps23 in the endosomal membrane and its binding to different molecules. **(B)** EhVps23 domains. The molecules that EhVps23 interacts with are marked below the annotated number of amino acid in the respective domains. **(C)** Western blot of extracts from trophozoites of: (WT) wild type strain (HM1:IMSS), Neo: transfected with the empty *pNeo* plasmid, Neo-EhVps23: transfected with the *pNeoEhvps23* plasmid, separated by 10% SDS-PAGE and blotted with α-EhVps23, or α-HA, or α-actin antibodies. **(D)** Densitometry of three independent western blots. **(E)** Confocal microscopy image of Neo-EhVps23 and Neo trophozoites using α-EhVps23 and α-HA antibodies. Scale bar = 10 μm. The squared area is magnified in **(F)**. Arrows: vesicles labeled by the protein. **(G)** Quantification of the fluorescence by pixel counting. ** P < 0.01, ***P < 0.001.

EhVps23 is a protein actively involved in the *E. histolytica* vesicular trafficking through the interaction with proteins of the ESCRT machinery and other molecules such as EhADH and EhVps32, EhUbiquitin and LBPA, among others (**Figure 1A**). To explore deeper the functions of this protein, we first generated trophozoites overexpressing the EhVps23 protein. We cloned into the *Neo* plasmid the *Ehvps23* full-length gene to produce the *pNeoEhvps23* construct (**Figure 1B**) labeled with HA. In western blot assays, α-HA antibodies developed a 54 kDa band that was also recognized by the α-EhVps23 antibody. Extracts of Neo-EhVps23 trophozoites grown with 40 µg/ml of gentamicin (Hamann et al., 1995) showed the band 50% stronger than the one developed in trophozoites transfected with the empty vector, used as control (**Figures 1C, D**). In concordance with western blot assays, immunofluorescence assays evidenced the exogenous protein enriched in vesicles close to the plasma membrane (**Figures 1E, F**), while trophozoites transfected with the empty vector gave no signal. Neo-EhVps23 trophozoites exhibited twice more pixels (α-EhVps23) than the control (**Figure 1G**).

**Figure 2:**
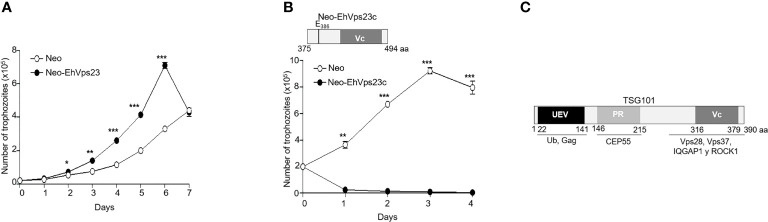
Growth curves of trophozoites transfected with **(A)** p*NeoEhvps23* and **(B)** *pNeoEhvps23*_1123-1485 bp_ plasmids. **(C)** Scheme of TSG101 protein domains and interaction with other proteins. *P < 0.05, **P < 0.01, ***P < 0.001.

Interestingly, growth curves evidenced that in the presence of 40 µg/ml of G418 Neo-EhVps23 trophozoites grew almost twice more rapidly than the control (**Figure 2A**). The trophozoites transfected with the construct *pNeoEhvps23*_1123-1485 bp_, which carries 362 bp (375-494 aa of the carboxy terminal) died in all experiments (**Figure 2B**), even at doses as low as 3 µg/ml of gentamicin. We hypothesized that the EhVps23-core polypeptide could capture molecules necessary for vital cell functions, including those involved in cell division, as reported for the human TSG101 protein (**Figure 2C**), (Morita et al., 2007), competing with the endogenous protein and causing a dominant negative effect. These experiments confirmed that, as it has been reported for mammalian transformed cells transfected with TSG101 (Liu et al., 2019), trophozoites transfected with EhVps23 grow faster.

### Electron Microscopy (SEM and TEM) Revealed Scarce Modifications in the External Morphology, and Abundant Helical Arrangements and Vacuoles Containing EhVps23 in Neo-EhVps23 Trophozoites

**Figure 3:**
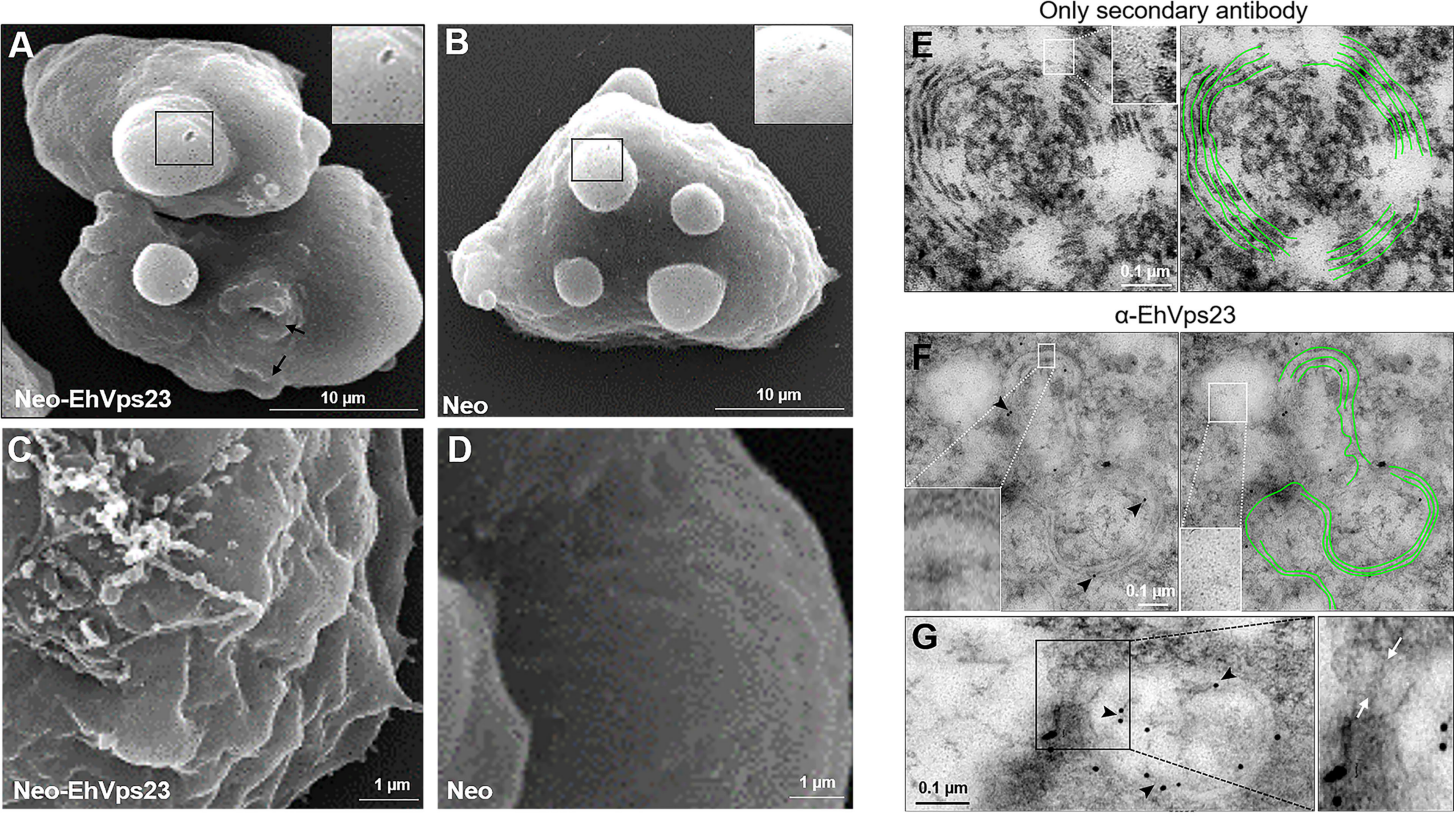
Scanning and Transmission Electron Microscopy of transfected trophozoites. Trophozoites were treated as described for SEM or TEM. **(A, B)** SEM of trophozoites transfected (10,000x) with the *pNeoEhvps23* **(A)** and *pNeo* **(B)** plasmids. Squared areas show part of the surface protuberance that are magnified at the right. Arrows: phagocytic cups. **(C, D)** Show part of the surface of trophozoites (25,000x). **(E–G)** TEM of thin sections of trophozoites transfected with the *pNeoEhvps23.* The helicoidal structures are remarked in green. **(F, G)** TEM images were obtained after treating the thin sections with α-EhVps23 followed by gold-labelled α-rat antibodies. Squared areas are magnified to show the very small vesicles in those regions. Arrowheads: label of the antibodies used to detect EhVps23. **(F)** The communication between vesicles where we visualized the presence of EhVps23 is shown. The magnification shows the communication channel between the vesicles.

To investigate how the overexpression of EhVps23 affected the morphology of the trophozoites, we performed electron microscopy experiments. SEM images of Neo-EhVps23 and control cells evidenced protuberances of distinct sizes with multiple pores in the surface (**Figures 3A, B**). The only difference that we detected between transfected and control trophozoites was that Neo-EhVps23 amoebae presented rougher surface and membrane ripples (**Figures 3C, D**), although we do not know yet if this is related to a cellular function involving EhVps23, as could be the secretion of molecules.

The thin sections prepared for TEM revealed helical structures in Neo-EhVps23 trophozoites. The control without primary antibodies is shown in **Figure 3E**. This structures are like the ones formed by the EhVps32 protein involved in the neck scission during ILVs formation (Avalos-Padilla et al., 2018). The α-EhVps23 and the gold-labelled antibodies recognized these structures (**Figure 3F**), suggesting that EhVps23 could participate in ILVs strangulation by activating ESCRT-III members, as it occurs in yeast and human (Alonso et al., 2011). Other possibility is that this protein could be carrying other molecules for secretion or transport them to other compartments. This later assumption is supported by the fact that magnification of the images evidenced large groups of vesicles distributed along these arrangements (**Figure 3G**). It is possible that these vesicles could be connecting among them, strengthening the hypothesis that EhVps23 could participate in the transport of molecules and, possibly, in cell-cell communication, as it has been reported for cancer cells (Harada et al., 2017; Nkosi et al., 2020).

**Figure 4:**
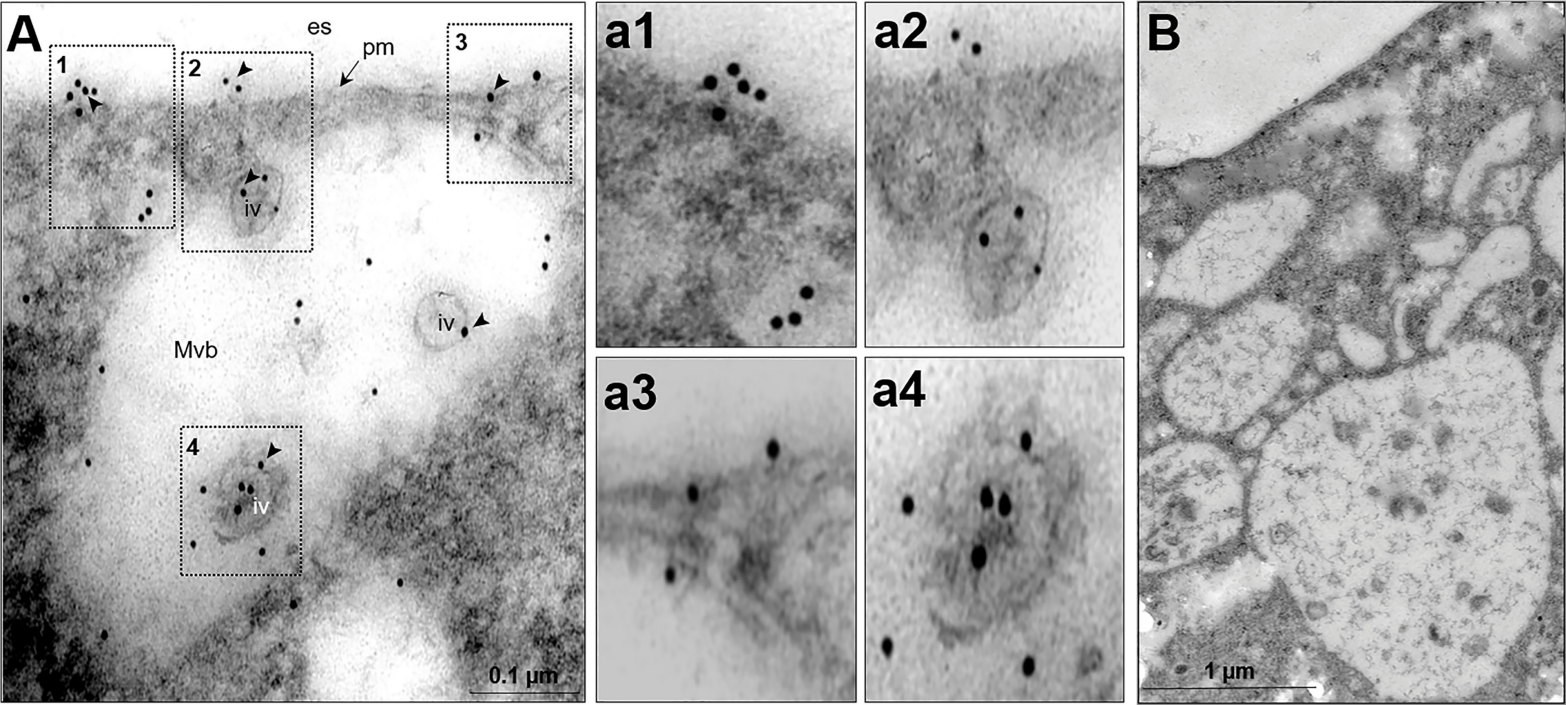
TEM of transfected trophozoites showing the EhVps23 in MVBs. **(A)** Thin sections of trophozoites treated with the α-EhVps23 followed by gold-labelled α-rat antibodies. Squared areas are magnified at right. **(B)** Control without using the first antibody.

Further examination of thin sections treated with α-EhVps23 and secondary gold-labelled antibodies, showed MVBs of about 551 nm of diameter, close to the plasma membrane, carrying vesicles, probably ILVs, of about 66-90 nm labeled by the antibodies (**Figure 4A**). Labeled vesicles of distinct size, appeared also outside the MVBs and in the extracellular space (**Figures 4A** a1-a4). No label was observed in the negative control (**Figure 4B**). Altogether, these results reinforce the hypothesis that the EhVps23 protein could be secreted.

### EhVps23 Is Secreted

**Figure 5:**
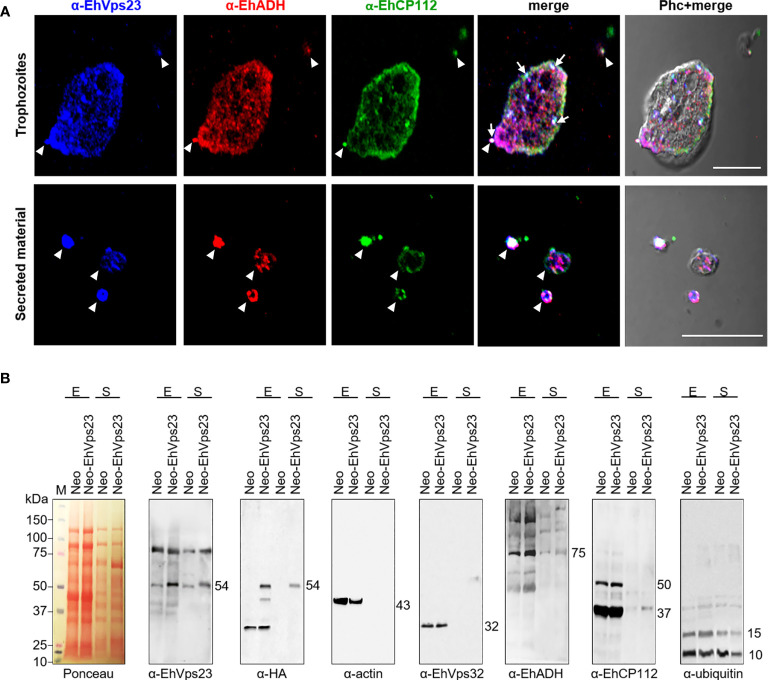
Immunodetection of EhVps23 in Neo-EhVps23 and Neo trophozoites and in the secreted products by trophozoites. **(A)** Confocal images of trophozoites and their secreted products treated with α-EhVps23, α-EhADH, α-EhCP112 antibodies and fluoresceinated secondary antibodies, according to the specie of each one of the antibodies used. Scale bar = 10 μm. **(B)** Ponceau stain and western blot of trophozoites extracts (E) and their secretion products (S) separated by SDS-PAGE and blotted with α-EhVps23, α-HA, α-EhVps32, α-EhADH, and α-Ubiquitin. α-EhCP112 was used as a positive control and α-actin as a control of the absence of trophozoites debris in the supernatants.

Immunofluorescence assays also revealed EhVps23 on the cell surface and in the extracellular space, colocalizing with the EhADH protein. Both proteins appeared concentrated in certain regions of the plasma membrane, in the cytoplasm, and in secreted material, together with the EhCP112 protease, that was used as positive control for secretion (Ocádiz et al., 2005; Bolaños et al., 2016) (**Figure 5A**).

To obtain further evidence on the secretion of EhVps23, we analyzed the supernatants of the trophozoites processed during secretion experiments (Bolaños et al., 2016). Proteins in the supernatants were separated by SDS-PAGE, transferred to nitrocellulose membranes and probed with specific antibodies and the corresponding secondary antibodies. The specific antibodies developed the EhVps23 protein in the cellular extracts and in the medium used to search secretion products, as described in Material and Methods. α-HA antibodies did not recognize the supernatants or the pellets of cell transformed with the *pNeo* empty vector, used as control (**Figure 5B**). Importantly, the α-actin antibodies did not detect any protein in the supernatants, indicating that no significant rupture of the cells occurred (**Figure 5B**). Additionally, we search for EhADH and EhVps32 proteins, both members of the ESCRT machinery. EhVps32 did not appear in the secretion products, although we do not discard that it suffered degradation during the experimental process. In contrast, EhADH was revealed as a protein that is also secreted, as well as the EhCP112 cysteine protease, used here as a positive control (Ocádiz et al., 2005; Bolaños et al., 2016) (**Figure 5B**). EhUbiquitin also appeared in the secretion products, probably bound to other proteins or as a free molecule (**Figure 5B**).

**Figure 6:**
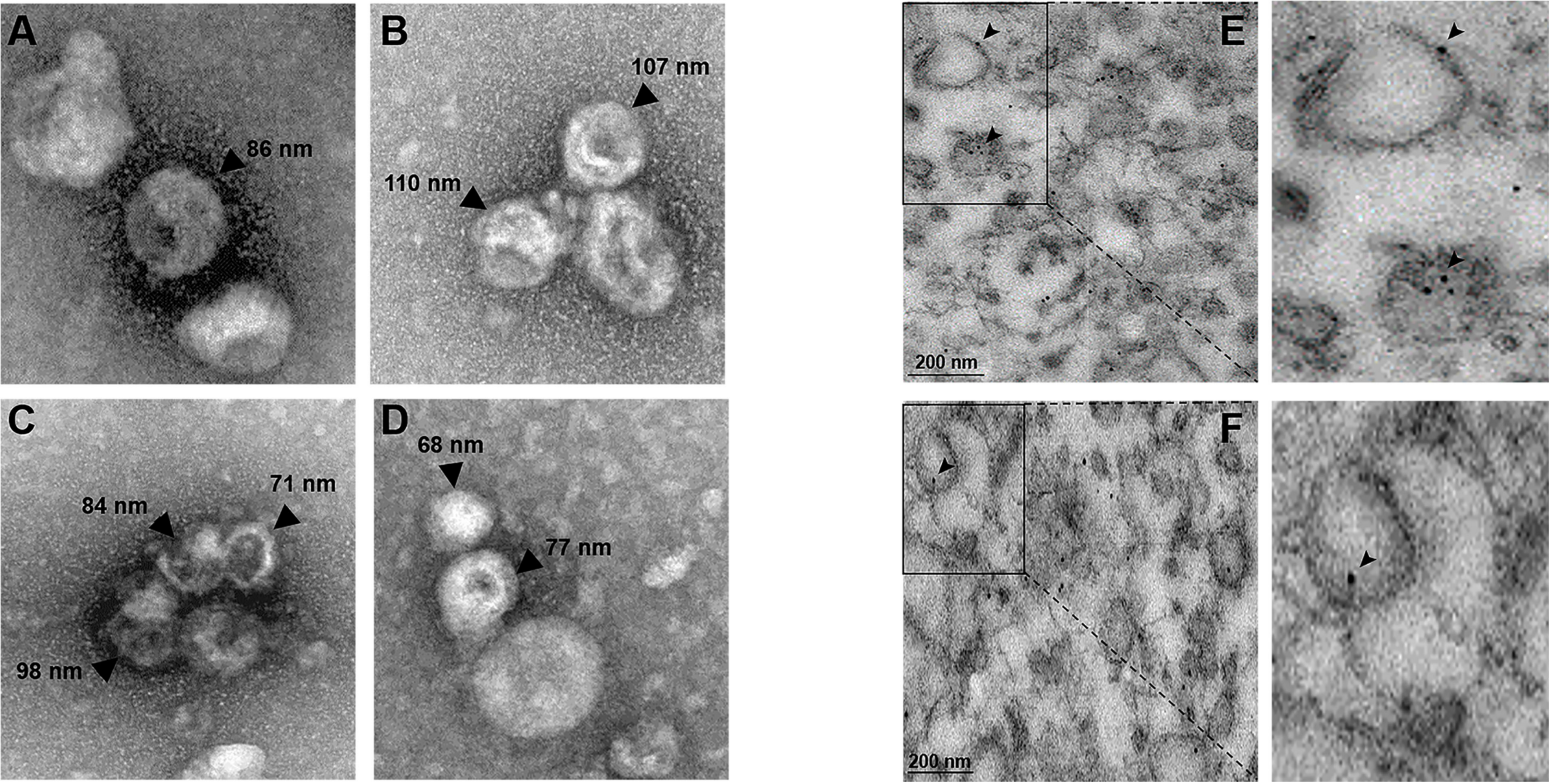
Transmission electron microscopy of vesicles found in secretion products. **(A–D)** Transmission electron microscopy of negative stained vesicles in the secretion products. The numbers indicate the different sizes calculated by the transmission electron microscope program JEM-1011. **(E, F)** TEM images (15 000X) of purified extracellular vesicles, the thin sections were incubated with α-EhVps23 followed by gold-labelled α-rat antibodies (10 nm). Squared areas are magnified to show EhVps23 protein in vesicles of different sizes. Arrowheads: label of the antibodies used to detect EhVps23. Scale: 200 nm.

Electron microscopy of the supernatants of secretion experiments, using a negative staining technique, exhibited vesicles of different size from 68 to 110 nm, with the appearance of exosomes (**Figures 6A–D**), giving further support to the hypothesis that EhVps23 is secreted together with other proteins. Furthermore, extracellular vesicles were purified from these secretion products and analyzed by TEM and labeling with anti-Vps23 antibody (**Figures 6E, F**). The EhVps23 protein is observed within the vesicles (**Figure 6E**) and near their plasma membrane (**Figure 6F**).

**Figure 7:**
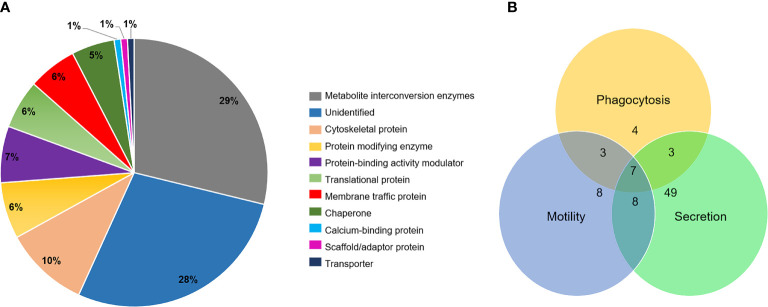
Graphical distribution of the proteins found in the immunoprecipitation of EhVps23 from total extracts of trophozoites by mass spectrometry. **(A)** Proteins class analysis of the immunoprecipitation of EhVps23 proteome showing predicted molecular functions. The chart was created using PANTHER GO analysis. **(B)** Analysis of the proteins involved in phagocytosis, motility and secretion.

Next, we investigated the proteins that interact with EhVps23 by immunoprecipitation experiments, using α-EhVps23 antibodies. Precipitated proteins were separated by gel electrophoresis and examined by mass spectrometry. Results of the analysis evidenced the presence of several proteins, from which we selected those related to vesicular trafficking (**Table 1**), phagocytosis (**Table 2**) (Okada et al., 2005), motility (**Table 3**) (Markiewicz et al., 2011) and secretion (**Table 4**) (Sharma et al., 2020) (**Figures 7A, B**). Some of them participate in more than one function, as it is shown in **Figure 7B**.

**Table 1:** Proteins associated with vesicular trafficking.

Protein	Access number
EhVps26	EHI_008470
GTP-binding protein (SAR1)	EHI_031410
EhVps35	EHI_041950
Ras family GTPase	EHI_058090
Rho family GTPase	EHI_068240
Rho family GTPase	EHI_070730
EhRab1A	EHI_108610
Rho family GTPase	EHI_129750
EhRabC3	EHI_143650
Rho GDP exchange inhibitor	EHI_147570
EhRabC1	EHI_153690
EhVps45A	EHI_160900
Rab family GTPase	EHI_164900
Rab GDP dissociation inhibitor alpha	EHI_167060
EhRabX11	EHI_177520
Rho family GTPase	EHI_181250
Clathrin heavy chain	EHI_201510
Clathrin heavy chain	EHI_201710
Clathrin heavy chain	EHI_201940

**Table 2:** Proteins associated with phagocytosis*.

Protein	Access number
Elongation factor 1-alpha	EHI_011210
Uncharacterized protein	EHI_016130
Cysteine proteinase 2	EHI_033710
Ras family GTPase	EHI_058090
Uncharacterized protein	EHI_069560
Rho family GTPase	EHI_070730
Cysteine proteinase 1	EHI_074180
EhUbI1	EHI_083270
LIM zinc finger domain containing protein	EHI_096420
Peptidyl-prolyl cis-trans isomerase	EHI_125840
Rho family GTPase	EHI_129750
EhRabC3	EHI_143650
p21-activated kinase	EHI_148280
EhRabC1	EHI_153690
EhRabX11	EHI_177520
Rho family GTPase	EHI_181250

*Okada et al., 2005.

**Table 3:** Proteins associated with motility**.

Protein	Access number
Pyruvate, phosphate dikinase	EHI_009530
Cysteine proteinase 2	EHI_033710
H(+)-transporting two-sector ATPase	EHI_043010
Ras family GTPase	EHI_058090
Rho family GTPase	EHI_070730
Protein disulfide isomerase	EHI_071590
Cysteine proteinase 1	EHI_074180
3-oxo-5-alpha-steroid 4-dehydrogenase domain-containing protein	EHI_076870
EhUbI1	EHI_083270
DEAD/DEAH box helicase	EHI_093900
Filamin 2, putative	EHI_104630
Myosin heavy chain	EHI_110180
Rho family GTPase	EHI_129750
Phosphopyruvate hydratase	EHI_130700
Calreticulin	EHI_136160
EhRabC3	EHI_143650
Aldehyde-alcohol dehydrogenase	EHI_150490
EhRabC1	EHI_153690
3-ketoacyl-CoA synthase	EHI_158240
EhVps45A	EHI_160900
90 kDa heat shock protein	EHI_163480
Grainin 1	EHI_167300
EhRabX11	EHI_177520
Rho family GTPase	EHI_181250
ATP-sulfurylase	EHI_197160
70 kDa heat shock protein	EHI_199590

**Marquay Markiewicz et al., 2011.

**Table 4:** Proteins associated with secretion***.

Protein	Access number
Pyruvate, phosphate dikinase	EHI_009530
40S ribosomal protein S8	EHI_009870
Xaa-Pro dipeptidase	EHI_010070
Elongation factor 1-alpha	EHI_011210
Leucine rich repeat protein	EHI_015120
Calmodulin	EHI_023500
Cysteine synthase A	EHI_024230
GTP-binding protein	EHI_031410
Polyadenylate-binding protein	EHI_033250
Cysteine proteinase 2	EHI_033710
GOLD domain-containing protein	EHI_038590
Vacuolar protein sorting 35	EHI_041950
H(+)-transporting two-sector ATPase	EHI_043010
Uncharacterized protein	EHI_047800
PNP_UDP_1 domain-containing protein	EHI_048740
Tubulin beta chain	EHI_049920
WD_REPEATS_REGION domain-containing protein	EHI_050550
Pyruvate:ferredoxin oxidoreductase	EHI_051060
AAA domain-containing protein	EHI_053020
Ras family GTPase	EHI_058090
EF-hand calcium-binding domain containing protein	EHI_060740
Glutamine--tRNA ligase	EHI_062700
Uncharacterized protein	EHI_069560
Inositol-3-phosphate synthase	EHI_070720
ADP-ribosylation factor 1	EHI_073470
Cysteine proteinase 1	EHI_074180
Long-chain-fatty-acid--CoA ligase	EHI_079300
Seryl-tRNA synthetase	EHI_092640
DEAD/DEAH box helicase	EHI_093900
Galactokinase	EHI_094100
Alpha-1,4 glucan phosphorylase	EHI_096830
Fructose-1,6-bisphosphate aldolase	EHI_098570
Epimerase domain-containing protein	EHI_098800
NAD(FAD)-dependent dehydrogenase	EHI_099700
Uncharacterized protein	EHI_101240
Filamin 2, putative	EHI_104630
EhRab1A	EHI_108610
Phosphoglucomutase	EHI_110120
Myosin heavy chain	EHI_110180
Uncharacterized protein	EHI_118750
Enhancer binding protein-1	EHI_121780
MHD domain-containing protein	EHI_124560
Rho family GTPase	EHI_129750
Phosphopyruvate hydratase	EHI_130700
Adenylate kinase	EHI_135470
Adenylyl cyclase-associated protein	EHI_136150
Rab family GTPase	EHI_143650
Thioredoxin domain-containing protein	EHI_145840
Aspartate--ammonia ligase	EHI_148470
Aldehyde-alcohol dehydrogenase	EHI_150490
Flavodoxin-like domain-containing protein	EHI_152650
Actin-related protein 2/3 complex subunit 4	EHI_152660
Uncharacterized protein	EHI_161070
Aspartyl-tRNA synthetase	EHI_175050
Profilin	EHI_176140
EhRabX11	EHI_177520
Acetyl-CoA synthetase	EHI_178960
Rho family GTPase	EHI_181250
Actin	EHI_182900
Glyceraldehyde-3-phosphate dehydrogenase	EHI_187020
Phosphoglycerate kinase	EHI_188180
ATP-sulfurylase	EHI_197160
ADF-H domain-containing protein	EHI_197480
Fe-ADH domain-containing protein	EHI_198760
Clathrin heavy chain	EHI_201510
Clathrin heavy chain	EHI_201710
Clathrin heavy chain	EHI_201940

***Sharma et al., 2020.

### Neo-EhVps23 Trophozoites Increment Their Rate of Phagocytosis

**Figure 8:**
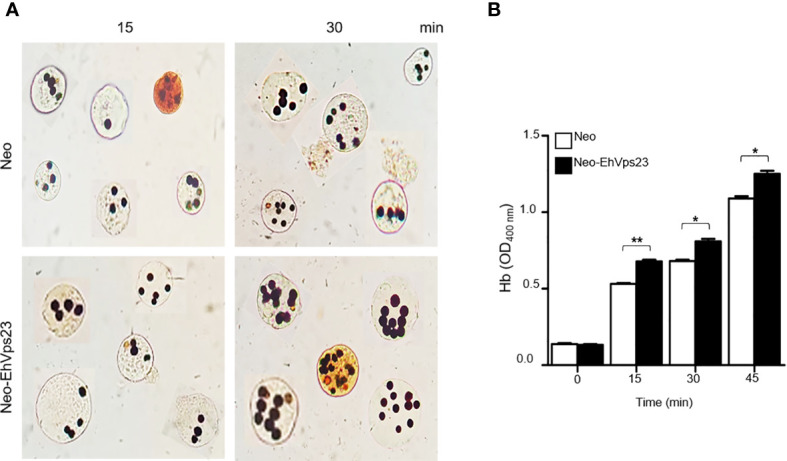
Phagocytosis of Neo-EhVps23 and Neo trophozoites. **(A)** Novikoff staining of erythrocytes ingested by the trophozoites after different times of RBCs incubation at 37°C. **(B)** Densitometry of the hemoglobin of the erythrocytes ingested. *P < 0.05, **P < 0.01.

Many authors, including us (García-Rivera et al., 1999), have reported that phagocytosis is one of the landmark virulence factors of amoebae. Immunofluorescence experiments, revealed that EhVps23 is closed to the erythrocytes since the initial contact, thus, EhVps23 could be involved in phagocytosis (Galindo et al., 2021). The rate of phagocytosis was measured by counting the ingested erythrocytes in 100 trophozoites and by the amount of hemoglobin inside the trophozoites (Novikoff et al., 1972; Talamás-Lara et al., 2014). Although no differences were detected at 5 min of phagocytosis in transfected cells, at longer times, Neo-EhVps23 trophozoites reproducibly increased 20% their rate of phagocytosis in comparison with the control (**Figures 8A, B**). The increment in the rate of phagocytosis of Neo-EhVps23 trophozoites evidenced the existence of a fine time and concentration control of the molecules that act in the distinct steps of phagocytosis.

### Neo-EhVps23 Trophozoites Increment Their Rate of Migration

**Figure 9:**
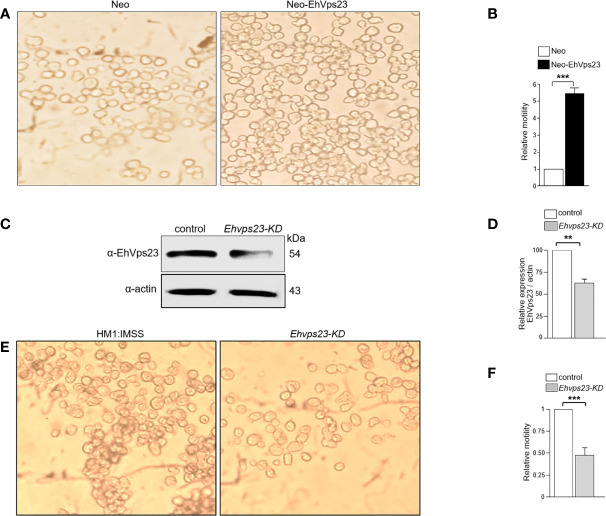
Motility of Neo-EhVps23 and *Ehvps23-KD* trophozoites. The trophozoites were placed in the upper chamber of Transwell filters and bovine serum in the lower chamber. After 3 h the trophozoites in the lower chamber was counted. **(A)** Neo-EhVps23 trophozoites found in the lower chamber of the Transwell filters. As control we used Neo trophozoites. **(B)** Quantification of trophozoites in the lower chamber to obtain the relative motility of Neo-EhVps23 trophozoites with respect to the control. **(C, D)** Western blot of silenced trophozoites (*Ehvps23-KD*) probed with the α-EhVps23 antibodies. Densitometry at the left using α-actin as control. **(E)** *Ehvps23-KD* trophozoites found in the lower chamber of the Transwell filters. As control we used trophozoites of the wild type strain HM1:IMSS. **(F)** Quantification of trophozoites in the lower chamber to obtain the relative motility of *Ehvps23-KD* trophozoites with respect to the control. **P < 0.01, ***P < 0.001.

Motility is a function widely involved in phagocytosis and invasion, both events require the emission of pseudopodia and the displacement of the prey inside the trophozoites. We analyzed the ability of Neo-EhVps23 trophozoites to move toward a chemoattractant, using Transwell filters (Bolaños et al., 2016). Trophozoites were placed in the upper chamber of the filters, and the lower chamber was charged with 500 μl of bovine serum. After 3 h at 37°C, Neo-EhVps23 trophozoites migrated five-fold faster than the control ones (**Figures 9A, B**), suggesting a role for EhVps23 in motility. This assumption was confirmed using trophozoites knocked-down in the *Ehvps23* gene (*Ehvps23KD* trophozoites) (**Figures 9C, D**), obtained as described (Solis et al., 2009; Galindo et al., 2021). These trophozoites exhibited a remarkable diminishing (more than 50%) in their ability to migrate toward the chemoattractant (**Figures 9E, F**). Results are in accordance with the reduction in the rate of phagocytosis showed by *Ehvps23KD* trophozoites reported recently (Galindo et al., 2021). Thus, the increase in the rate of phagocytosis and the faster movement exhibited by Neo-EhVps23 trophozoites, plausibly given by an EhVps23 excess in the cells, correlate with the effect also attributed to TGS101 protein in transformed cells (Chua et al., 2019; Liu et al., 2019). These results evidenced again similarities between amoebae overexpressing EhVps23 and transformed cells.

### Neo-EhVps23 Trophozoites Increase *In Vivo* Virulence

**Figure 10:**
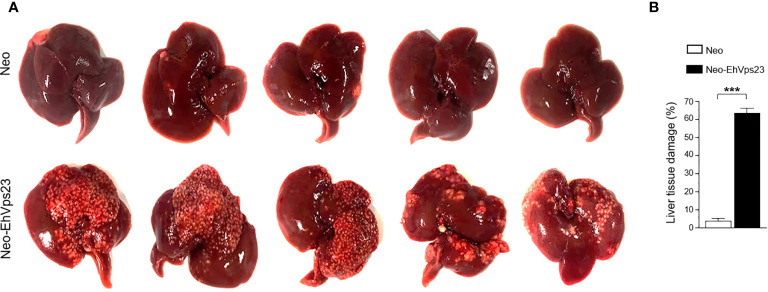
Livers of hamsters intraportally inoculated with Neo and Neo-EhVps23 trophozoites. **(A)** Animals under anesthesia were inoculated with Neo or Neo-EhVps23 trophozoites. Six days later, trophozoites were anesthetized and livers were extracted to examine the damage produced by the trophozoites. **(B)** Percentage of the damage was calculated by weighting the livers, weighting the damage tissue and obtaining the relation between them. ***P < 0.001.

The phagocytosis- and motility-increment in trophozoites overexpressing EhVps23, as well as the phagocytosis- and motility-diminishing in *Ehvps23KD* trophozoites, led us to hypothesize that EhVps23 could have a role in the *in vivo* virulence of trophozoites. To explore this, we investigated the capacity of Neo-EhVps23 trophozoites to produce hepatic abscesses in hamsters. After six days of inoculation, the animals looked sick. Thus, under deep anesthesia of the animals, we examined their livers. Neo-EhVps23 trophozoites produced a huge number of abscesses, whereas the Neo trophozoites, used as control, caused only few small ones (**Figure 10A**). Quantification of the healthy and damaged tissue showed that Neo-EhVps23 trophozoites damaged approximately 63% of the liver tissue, while hamsters inoculated with the same amount of Neo trophozoites, presented only 4% tissue damaged (**Figure 10B**). The damage presented by Neo-EhVps23 trophozoites could be related to the increase in phagocytosis, migration and growth rate showed by these parasites.

## Discussion

In this paper, we studied the functions performed by the EhVps23 protein, the unique member of the ESCRT-I complex detected until now in this parasite (Galindo et al., 2021). The study of EhVps23 calls our attention because: i) As an orthologue of the TSG101 protein, we were interested in investigating whether it was able to perform functions in the trophozoites such as those that have been adjudicated to TSG101 (faster growth, motility and invasion capacity), a protein involved in malignant transformation (Chua et al., 2019; Liu et al., 2019). The distinct populations of *E. histolytica* exhibit different degree of *in vitro* and *in vivo* virulence (Flores-Romo et al., 1997; Feng et al., 2020), and it is well known that under certain conditions, as several passages in culture after their isolation from a patient, the trophozoites attenuates their capacity to destroy tissues and invade organs (Luo et al., 2013). This fact evokes the events suffered by mammalian cells during malignant transformation. ii) Our interest in understanding processes involved in an infection that affects 50 million people and kills 100,000 each year around the world led us to characterize the EhVps23 protein, actively involved in vesicular trafficking, which at the time, influences several cellular functions, including virulence. iii) The relevance of our work is sustained by the facts that: it provides new evidence on the participation in virulence of a protein that had not been characterized until know. It gives evidence for reinforcing the role of the ESCRT machinery in phagocytosis and tissue invasion by the trophozoites. iv) The motility experiments using trophozoites that overexpress or poorly express the protein showed the close relation among the trophozoites motility, their rate of phagocytosis, and its ability to invade tissues. The knowledge of these functions and the molecules involved on them will help to discover their interconnections and those cellular events that make this parasite a professional pathogen. Hence, the molecular characterization of these proteins could provide tools to combat this parasitosis. v) Finally, the characterization of novel virulence molecules in *E. histolytica* is a logical continuation of our research on phagocytosis and cytopathic effect produced by trophozoites.

Trophozoites overexpressing the EhVps23 protein (Neo-EhVps23) present helical arrangements in the cytoplasm, where the protein was located, similar to the ones formed by EhVps32 for vesicle scission (Avalos-Padilla et al., 2015; Avalos-Padilla et al., 2018), suggesting that the protein participates in this event. However, we did not observe a close contact between EhVps23 and EhVps32 (Galindo et al., 2021). Both proteins could have an indirect interaction by means of other proteins of the ESCRT-III complex, or by the EhADH adhesin that binds to EhVps32 (Bañuelos et al., 2012), or as we said above, EhVps32 is degraded during the experimental procedures. Although molecular docking analysis predicted that EhVps23 binds to EhUbiquitin, EhADH and EhVps32 through the UEV domain (Galindo et al., 2021), the functional importance of the Vps23 core domain was evidenced by its lethal effect on trophozoites when transfected as a truncated protein. The overexpression of the EhVps23*_375-494 aa_* produced a dominant negative effect leading cells to death. The overexpression of this region could be stabilizing certain protein complexes or sequestering not identified proteins belonging to the ESCRT-I complex, as it occurs in other organisms (Kostelansky et al., 2007; Flower et al., 2020), including proteins necessary for the generation of MVBs and ILVs, indispensable in vesicular traffic events, endocytosis, and other functions (Vietri et al., 2020). In addition, it is possible that, as in other models, this region participates in the recruitment of proteins required for cell division (Morita et al., 2007). Therefore, when this region is overexpressed, key proteins for this event are sequestered, which is reflected in the death of the trophozoites.

By confocal and transmission electron microscopy, EhVps23 was detected in MVBs, vesicles, and in the extracellular space. This is consistent with the fact that the protein is secreted in exosomes-like vesicles together with other proteins, as it was recently shown by Sharma et al. (2020). Interestingly, the EhADH adhesin was also found there, although this protein was not detected by previous analysis (Sharma et al., 2020). We speculate that these abundant vesicles could carry molecules that participate in the prey capture or in cell-cell communication.

In conclusion, the study of cellular functions, revealed that Neo-EhVps23 cells showed 50% faster growth than the control, presented a significant higher rate of phagocytosis, and migrate five-fold faster than the control, in concordance with the low rate of migration exhibited by the knocked-down trophozoites. In addition, Neo-EhVps23 trophozoites produced dramatic liver abscesses in experimental animals. Altogether, our results showed that EhVps23 overexpression increases the virulence of trophozoites, resembling cancer cells that overexpress TSG101.

## Data Availability Statement

The raw data supporting the conclusions of this article will be made available by the authors, without undue reservation.

## Ethics Statement

The animal study was reviewed and approved by The Institutional Committee for Animal Care and Use (IACUC/Ethics committee) from CINVESTAV, Protocol Number 0505-12, CICUAL 001. Written informed consent was obtained from the owners for the participation of their animals in this study.

## Author Contributions

AG: research, methodology, and writing. RJ-R: research, methodology, supervision, and writing. GG-R: methodology and supervision. CB: supervision and writing. BC-M and LS-V: methodology. EO: supervision, writing, and research. All authors contributed to the article and approved the submitted version.

## Funding

This work was supported by the National Council for Science and Technology (Conacyt) of Mexico (grant A1-S8380 for EO), and RJ-R received a Conacyt Postdoctoral Fellowship.

## Conflict of Interest

The authors declare that the research was conducted in the absence of any commercial or financial relationships that could be construed as a potential conflict of interest.

## Publisher’s Note

All claims expressed in this article are solely those of the authors and do not necessarily represent those of their affiliated organizations, or those of the publisher, the editors and the reviewers. Any product that may be evaluated in this article, or claim that may be made by its manufacturer, is not guaranteed or endorsed by the publisher.
